# Phenotypic Plasticity of Nest-Mate Recognition Cues in *Formica exsecta* Ants

**DOI:** 10.1007/s10886-019-01103-2

**Published:** 2019-09-02

**Authors:** Stephen J. Martin, Falko P. Drijfhout, Adam G. Hart

**Affiliations:** 1 School of Environment and Life Sciences, Salford University, Manchester, M5 4WT UK; 2 Chemical Ecology Group, School of Chemical and Physical Sciences, Keele University, Newcastle, ST5 5BG UK; 3 School of Natural & Social Sciences, University of Gloucestershire, Cheltenham, GL50 4AZ UK

**Keywords:** Phenotypic variation, Chemotypes, Chemical plasticity, *Gestalt* model

## Abstract

It is well established that many ant species have evolved qualitatively distinct species-specific chemical profile that are stable over large geographical distances. Within these species profiles quantitative variations in the chemical profile allows distinct colony-specific odours to arise (chemotypes) that are shared by all colony members. This help maintains social cohesion, including defence of their colonies against all intruders, including con-specifics. How these colony -level chemotypes are maintained among nest-mates has long been debated. The two main theories are; each ant is able to biochemically adjust its chemical profile to ‘match’ that of its nest-mates and or the queen, or all nest-mates share their individually generated chemical profile via trophollaxis resulting in an average nest-mate profile. This ‘mixing’ idea is better known as the *Gestalt* model. Unfortunately, it has been very difficult to experimentally test these two ideas in a single experimental design. However, it is now possible using the ant *Formica exsecta* because the compounds used in nest-mate recognition compounds are known. We demonstrate that workers adjust their profile to ‘match’ the dominant chemical profile within that colony, hence maintaining the colony-specific chemotype and indicates that a ‘*gestalt*’ mechanism, i.e. profile mixing, plays no or only a minor role.

## Introduction

Eusocial insects are among the most abundant of the terrestrial arthropods and of major ecological importance in most terrestrial ecosystems. Recognition between units is a fundamental characteristic of all biological systems across all levels of organisation (Wyatt 2003). However, in social insects a ‘recognition unit’ is often the colony, which consists of tens, thousands or even millions of individual workers. In large colonies it is unlikely that workers could learn every individual odour. A simpler solution is for all nest-mate to have the same odour, thus preserving the integrity of the colony. This ensures that all altruistic behaviours of workers (e.g. nest defence and caring for the queen’s offspring) are directed towards their relatives. Consequently, understanding how a common odour is maintained is a critical aspect of sociality.

Most ant species have a unique species-specific qualitative blend of cuticular hydrocarbons (CH) (Martin and Drijfhout 2009) within which a quantitative change of a sub-set of CH’s are colony-specific (Martin et al. 2008). Despite extensive research just how variation in these colony chemotypes is maintained has remained a mystery, since individual genetic variations in odour produced by each nest-mate needs to be unified to create a common colony odour. The *Gestalt* model (Crozier and Dix 1979) could create a common colony odour (via social grooming (allogrooming) and social feeding (trophallaxis) (Boulay et al. 2000). However, it has long been suggested that individuals may modify their own profile in order to *match* a new environment (e.g. Bonavita-Cougourdan et al. 1989; Provost et al. 1993). That is an individual adjusts its biochemical processes to modify its own cuticular profile to match that of its nestmates. However, it has been experimentally challenging to test these two ideas in a single experimental design, since the sub-set of CH’s used in recognition were unknown. However, in the ant *Formica exsecta* the nest-mate recognition compounds are known (Martin et al. 2008). Therefore, the aim of this study was to conduct a single experimental design using *F. exsecta* workers to test if the unified nest-mate profile arises through a ‘matching’ or ‘mixing’ mechanism.

## Methods and Materials

### Study Species

**Fig. 1:**
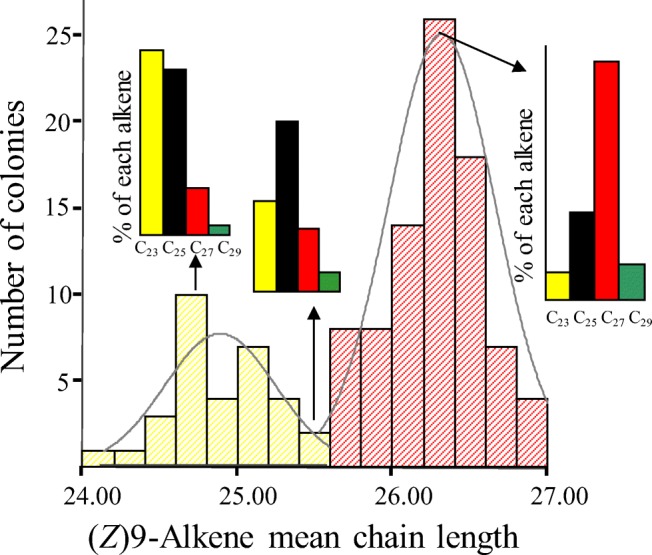
The distribution of 117 *Formica exsecta* colony chemo-types from the Tvärminne zoological station area. A single mean chain length (MCL) is calculated for each colony using their (*Z*)9-alkene profile. Each colony profile was determined from the individual analysis of 10 nest-mates. The (Z)-9-alkene profile ranges from colonies being C_23:1_ dominant (yellow-hatch) to C_27:1_ dominate (red-hatch), as illustrated by three actual colony profiles, which in this population forms a bi-modal distribution

Previous chemical analysis of workers from 117 *F. exsecta* colonies located within a 30 km radius of the Tvärminne zoological station in Hankö, Finland, revealed distinct colony-specific Z9-alkene profiles (henceforth referred to as “chemotypes”) that ranged from C_21:1_ to C_27:1_ (Fig.1; Martin et al. 2013). We focused solely on the four Z9-alkenes (C_21:1_, C_23:1_, C_25:1 &_ C_27:1_), which have been shown to be the only active CH in nest-mate behavioural bioassays and form the colony-specific profile in this species (Martin et al. 2008).

### Mixing or Matching Experiments Using *Formica exsecta* Workers

During the summer of 2010 and 2011 colony fragments containing ~100 workers, and natal nest material were collected from 24 colonies. In 2011 up to 50 worker callows i.e. newly emerged workers that are pale in colour, were also collected. Each colony fragment (ants + material) was maintained in a plastic box at 15 °C in a 12:12 light/dark regime for a week prior to the experiment. Fragments were provided with water and fed weekly with a diet of egg, honey and agar. These fragments were chosen using existing chemical data so that their colony chemotypes were either C_23:1_ or C_27:1_ rich, i. e. lie at the opposite ends of the chemotypes present in the Finnish population (Fig. 1). This was confirmed by the Gas Chromatograph-Mass Spectrometer (GC-MS) analysis (see below) of ten workers from each fragment at the start of the experiment and represents the natal colony profile. In total, 12 fragments were C_23:1_ rich (yellow) and 12 fragments were C_27:1_ rich (red). In the Tvärminne area greater than 90% of colonies are monogynous i.e. headed by a single queen. All study colonies possessed traits typical of monogynous colonies, i.e. large workers, small distinct mounds ect.

From these fragments, 22 new ‘host’ colonies each containing 20 nestmate workers were established in pots whose sides were sprayed with Soudal Silicone spray to prevent escape. Each pot was 7 cm in diameter, had a plaster base that was kept damp, and contained no nest material, to prevent any non-ant nest-specific chemical cues being present. All colonies were maintained at a constant 15 °C in a 12:12 light dark regime. These consisted of 14 C_23:1_ rich (yellow) and eight were C_27:1_ rich (red) host colonies. After a 24-h settling-in period, two adult or (callow) ants (the “alien” workers) were removed from their natal fragment and added into the host colony. The alien workers were introduced into a host colony with the opposite profile i.e. C_23:1_ into C_27:1_ and vice-versa, except for the nine controls where the host and alien ants where from different colonies but had similar profiles i.e. C_23:1_ into C_23:1_ and C_27:1_ into C_27:1._ Callows are pale allowing them to be easily identified from host workers during the length of the experiment, but the adult alien workers were marked with a small amount of coloured water-soluble ink from a POSCA™ pen to allow identification. We have already established that marking ants in this way does not affect ant behaviour (Martin et al. 2012b), or their CH profile. Two days later another two ants (of the same class as before: callow or adult) from the same natal colony were added into the host colony, and these additions continued until, after 20 days, each colony contained 20 unmarked host and 20 marked adult or 20 callow, alien ants. The colonies were then maintained for a further 21 days, being fed weekly on a standard diet before being freeze-killed at −80 °C. During the 21-day post-introduction period any dead ants were replaced, or host ant removed, in order to maintain a balanced experimental design. That is, equal numbers of both host and alien ants present, so we can expect any mixed profile will be midway between that of the host and alien natal colony profiles.

This experimental set up is possible since, although *F. exsecta* workers are highly aggressive when defending their colonies, in neutral areas such as the pots in which they were housed aggression towards non-nestmates and callows is greatly reduced. Intense interactions were sometimes seen when ants where initially introduced, occasionally resulting in aggression and death, but after one day interactions between alien and host ants became non-aggressive and the host colonies appeared integrated.

No spatial separation between the introduced and host ants were seen, and appear to mix equally, as the marking of one group, or different colour of the callows, would have made any spatial separation easy to detect.

The progressive introduction of alien ants, during the first 20 days, means if ‘*mixing*’ is occurring the dominant chemotype becomes less dominant as the experiment progressed until at the end of the experiment when the number of host and alien ants are equal, then the average profile will be midway between the two natal colonies. If ‘*matching’* was occurring, then each introduced alien ant would change its profile towards that of the dominate chemotype i.e. the hosts. Furthermore, the hosts should remain unchanged throughout the experiment since it is the introduced ants that are always in the minority.

### Chemical Analysis

Ants were stored at -80 °C prior to chemical analysis. Each worker was placed in a glass vial with 50 μl of HPLC-grade hexane, containing 10 μg ml^−1^ of an internal standard (docosane, C_20_ alkane), for 1 h at -80 °C, as this allowed RNA to be extracted. We had previously established that this extraction method did not affect the subsequent CH profile. Ants were removed, the hexane was evaporated in air at ambient temperature and the dry extracts were stored at −20 °C until required for analysis. Samples were analysed on an Agilent 7890 gas chromatograph (GC) connected to an Agilent 5975 MSD (quadropole) mass spectrometer (MS; –70 eV, electron impact ionization). The GC was equipped with a ZB-5HT column (30 m length; 0.32 mm ID; 0.25 μm film thickness), and the oven temperature was programmed from 50 °C to 110 °C at 40 °C min^−1^ and then from 110 °C to 360 °C at 20 °C min^−1^. Samples were injected in splitless mode, with helium as the carrier gas, at a constant flow rate of 1.0 ml min^−1^. Z9-alkenes were characterized using diagnostic ions and their Kovats indices. We rejected runs if the total ion count did not exceed 10^7^, due to the inaccuracy of integrating small peaks (personal observation).

Using the total ion chromatogram, the peak area of each Z9-alkene was manually integrated. The proportion of each of the four key Z9-alkenes (C_21:1_, C_23:1_, C_25:1_ & C_27:1_) was calculated relative to the total amount of these four Z9-alkenes. These data were then used to calculate a Z9-alkene mean chain-length for each ant as follows:

$$ \mathrm{Mean}\ \mathrm{chain}-\mathrm{length}=\left(\Sigma \left(\%{\mathrm{C}}_{n:1}\ \mathrm{x}\ n\right)\right)/100 $$

where *n* is the chain length. Regression of mean chain-length (MCL) against 117 colony profiles indicated that MCL accounted for 83% of the overall variation (Martin et al. 2012b) since colony differences are contained predominantly in the changing ratios of C_23:1_ and C_27:1_ (Martin et al. 2008, 2013, Fig. 1). Although it is theoretically possible for two ants to have a different profile but the same MCL, this cannot happen in this system since each nestmate CH profile always has a bell-shaped distribution and hence are centred around a mean, meaning that MCL is an excellent way to capture the variation within this system.

## Results

**Fig. 2:**
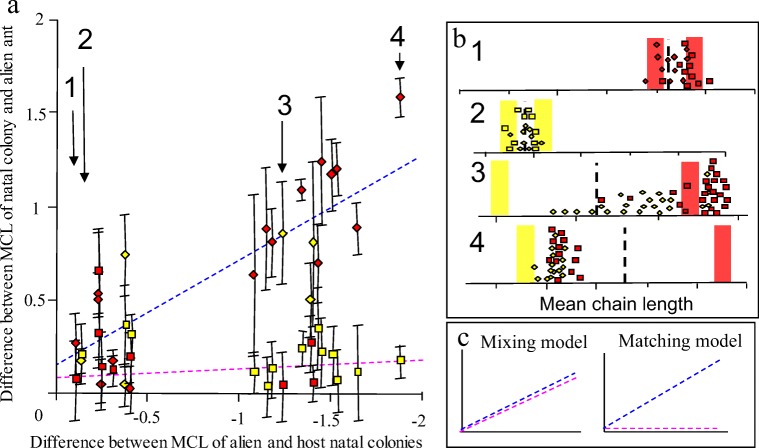
**Tests if the experimental data supports the mixing or matching model**. a) It plotted the data from the 22 colonies (mean + SD) depending on the chemical distance between alien and host natal colonies (x-axis), and how much the alien ant had changed measure by the difference between the natal colony and alien ant (y-axis). Alien ants (diamonds) and host ants (squares) are coloured yellow or red indicating a C_23:1_ or C_27:1_ rich natal colony, respectively. b) Illustrates data from the four different options (see Fig. 3) and the numbers in (a) indicate the position of the colony data on the main graph. c) Shows the two possible outcomes; In a mixing model the change in alien ants (blue) always equals the change in host ants (pink), whereas, in a matching model, only the alien ants (blue) would change, not the host ants (pink). As the starting chemical distance between the host and alien ants increases so does the amount of change required in the alien ants

**Fig. 3:**
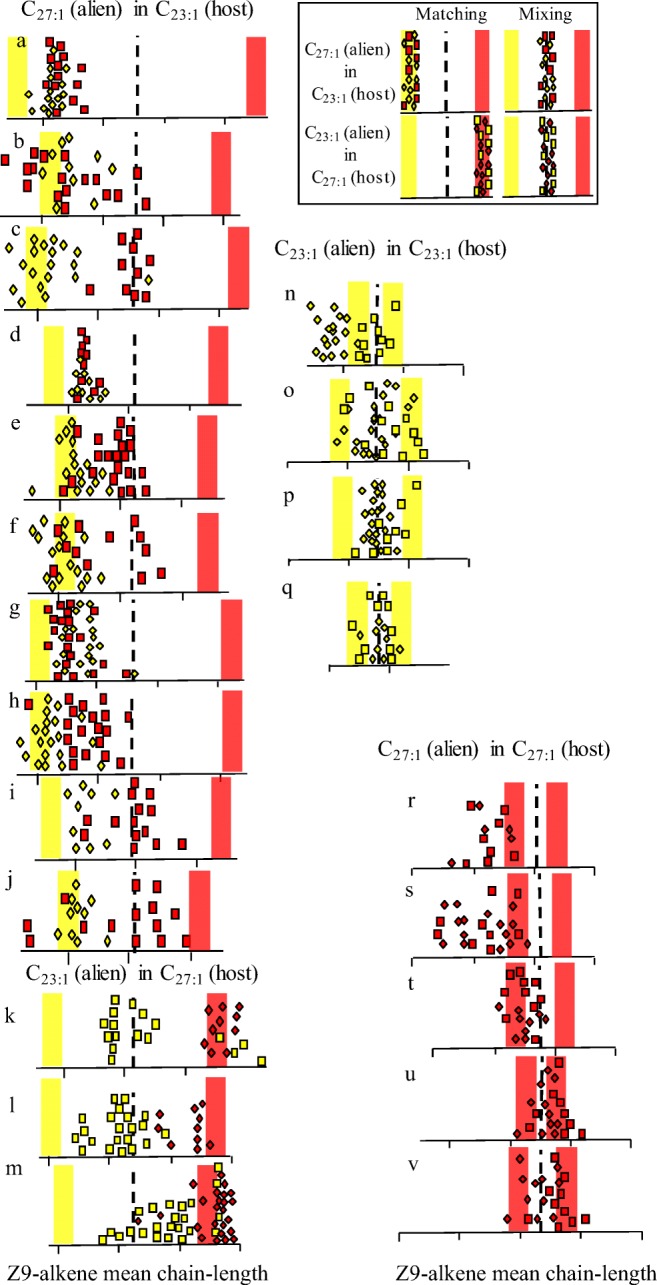
The results from the 13 (a-m) experimental and nine control (n-v) colonies, where 20 host ants with a C_23:1_ rich (yellow) or C_27:1_ rich (red) chemotype were mixed with 20 alien ants of the opposite chemotype, or the similar chemotype in the controls. The original mean chain-length of the natal chemotype is represented by the red or yellow bars. The resulting profile at the end of the experiment for each introduced alien (square) or host (diamond) ant is shown. The introduced aliens were either callows (a-f, k) or adults (g-j, l, m). The dotted line indicates the predicted mixed profile based on the two starting chemotypes. The insert indicates the predicted outcome under perfect matching or mixing

A total of 611 high quality chromatograms were analysed from the 22 experimental colonies. A minimum of ten host and ten alien ants were analyzed from each experiment. In every colony the Z9-alkene profile of the host ants remained similar to that of their original, natal, colony, whereas the introduced alien ants altered their profile towards that of their hosts’ profile (Fig. 2). The greater the chemical distance between the natal colonies resulted in a corresponding greater change observed in the alien ants (Fig. 2). These changes in CH profile occurred in both directions i.e. C_23:1_ alien ant shifted to match C_25:1_ host and vice-versa (Fig. 3). Whereas, in the controls where host and alien workers had similar profiles, no shifts in chemotype occurred (Fig. 3), confirming that being placed into an alien colony did not on its own, affect alkene profile.

## Discussion

In all 13 (Fig. 3) experiments the profiles of the alien *F. exsecta* workers moved away from their natal colony profile towards that of their host colony’s profile. Conversely, the host profiles always remained similar to that of their natal colony (Fig. 3). Only a matching model can explain the data generated by this study since the *gestalt* model predicts a mixing of profiles to some intermediate profile. This finding is almost identical to that of Carlin and Holldobler (1983), who used mixed species colonies of *Camponotus* ants to show that based on aggression tests, a ‘queen discriminator’ (matching) hypothesis, i.e. workers matching their host queens’ odour was supported and mixing (*gestalt*) theory rejected. Their mixed colonies were generated by the introduction of varying proportions of alien larvae into the host nest, and we now know that larvae of the ant *Camponotus vagus* can modify their CHC profile to match that of an unrelated colony, to the point of becoming identical to those of the larvae of the host colony (Bonavita-Cougourdan et al. 1989).

Yamaoka (1990) showed that the queen was crucial to maintaining a consistent colony odour among nestmates and as all colony fragments where queen-less, an increased variation of nestmates profiles was expected, and seen (Fig. 3). We have shown that the presence of a queen is not necessary for profile matching to occur, but her presence would be predicted to accelerate the matching process as demonstrated by (Provost et al. 1993). This makes biological sense as a founder queen’s profile can be converted into a colony profile since each new worker has the ability to ‘match’ its profiles to that of their much larger queen’s chemotype and nestmates. As the colony grows each new batch of workers will continue to match their profile to that the dominant colony odour, resulting in the formation and maintenance of the unique colony chemotypes generated by the queen and we see in the field (Fig. 1., Martin et al. 2013).

There will be physiological limits on how far matching is possible particular between species, which can have qualitative differences (Martin and Drijfhout 2009). This may explain why when two species share a common nest (parabiosis), both species retain their own distinct chemotypes (Errard et al. 2003; Menzel et al. 2009; Emery and Tsutsui 2013) rather than generate a common ‘mixed’ nest odour. The same principle may apply where enslaved ants maintain their own species profile (Errard et al. 2006) in their new host colony. This may also explain why there are very few clear cases where parasites are known to mimic (match) ‘by synthesis’ the recognition compounds of other species e.g. the termite nest occupying staphylinid beetle *Trichopsenius frosti* (Howard et al. 1980), parasitic Maculinea butterflies (Akino et al. 1999) and *Psithyrus* bumblebees (Martin et al. 2010).

It is now clear that some ants (Sharma et al. 2015; Slone et al. 2017) and honeybees (Chaline et al. 2005) can detect individual hydrocarbons by chain length with antennal receptors. By using specialized biochemical controls, probably involving the elongase system in hydrocarbon production (Morgan 2010; Wicker-Thomas and Chertemps 2010), it would be possible for ants to adjust the chain length of the hydrocarbons produced to match the length of those of its more numerous hosts. There is also evidence that the external microbes may also be used in nestmate recognition in ants (Dosmann et al. 2016) and termites (Matsuura 2001). However, as we only observed changes in the Z9-alkene profiles in the host ants placed in alien hosts with a different Z9-alkene profile, and not in the controls despite the ants coming from different colonies, so implying different micro-biome. Therefore, additional studies are needed to determine fully how changes in host-microbe interactions may be involved with CHC production (Vuong et al. 2017).

This study supports previous research on *F. exsecta* (Martin et al. 2012a, b) and *F. fusca* (Helanterä et al. 2011) that shows that greater genetic diversity does not generate a broader colony odour or recognition template. In fact, the opposite occurs, at least in *Formica* ants. Guerrieri et al. (2009) and others have suggested that ants recognize non-nestmates rather than nestmates, but *F. exsecta* ants must be aware of the differences between their own and their nestmates’ Z9-alkene profile in order to continually adjust it to match that of the majority profile. This means that any internal template will be much more dynamic than previously assumed (e.g. Newey 2011; Esponda and Gordon 2015). In *F. exsecta* aggression levels are highly variable when the individual Z9-alkene profiles (recognition cues) are small but undergo a step change beyond a threshold where all variability in behavior disappears and aggression is always high (Fig. 3, Martin et al. 2012a). This was observed in ants from both monogynous and polygynous colonies, but differences in the *n*-alkanes profiles did not affect aggression levels, indicating the importance of knowing the actual recognition compounds. This type of individual variation in sensitivity, assumed by the previous models (e.g. Newey 2011; Esponda and Gordon 2015), can only be seen when chemical differences between ants were small, since, if the differences were large, all ants could perceive them. A similar situation occurs in the hygienic response in honeybees, where age-related changes in neuromodulator octopamine levels in the brain heighten their perception to dead or dying brood for a period of just five days (Spivak et al. 2003). A potentially similar phenomenon is known in ants (Kostowski et al. 1975; Muscedere et al. 2011) and may explain the findings of Newey et al. 2010 on weaver ants (*Oecophylla smaragdina*) and task dependent aggression in the red harvester ant (*Pogonomyrmex barbatus*) (Sturgis and Gordon 2013), where individuals of different age (task) have different levels of perception.

In light of this study we need to reappraise the role of the ‘*gestalt*’ mechanism proposed by Crozier and Dix (1979), since it cannot unambiguously account for the findings of numerous studies. Furthermore, this type of phenotypic plasticity could challenge the importance of the notion that phenotype signals in both social and solitary (Otte et al. 2018) insects are an honest reflection of genotype since there use in kin-recognition would be somewhat error-prone (Carlin and Holldobler 1983).
